# The Mouse Genome Database: integration of and access to knowledge about the laboratory mouse

**DOI:** 10.1093/nar/gkt1225

**Published:** 2013-11-26

**Authors:** Judith A. Blake, Carol J. Bult, Janan T. Eppig, James A. Kadin, Joel E. Richardson, The Mouse Genome Database Group

**Affiliations:** Bioinformatics and Computational Biology, The Jackson Laboratory, 600 Main Street, Bar Harbor, ME 04609, USA

## Abstract

The Mouse Genome Database (MGD) (http://www.informatics.jax.org) is the community model organism database resource for the laboratory mouse, a premier animal model for the study of genetic and genomic systems relevant to human biology and disease. MGD maintains a comprehensive catalog of genes, functional RNAs and other genome features as well as heritable phenotypes and quantitative trait loci. The genome feature catalog is generated by the integration of computational and manual genome annotations generated by NCBI, Ensembl and Vega/HAVANA. MGD curates and maintains the comprehensive listing of functional annotations for mouse genes using the Gene Ontology, and MGD curates and integrates comprehensive phenotype annotations including associations of mouse models with human diseases. Recent improvements include integration of the latest mouse genome build (GRCm38), improved access to comparative and functional annotations for mouse genes with expanded representation of comparative vertebrate genomes and new loads of phenotype data from high-throughput phenotyping projects. All MGD resources are freely available to the research community.

## INTRODUCTION

The Mouse Genome Database (MGD) is the community model organism database resource for the laboratory mouse, a premier animal model for the study of genetic and genomic systems relevant to human biology and disease. Initially designed and implemented in 1994 to track genetic mapping data and to report on and describe mouse mutant phenotypes, MGD has grown to be the recognized authority for knowledge about mouse genes and as a comprehensive data integration site and repository for mouse genetic, genomic and phenotypic data derived from primary literature as well as from major data providers (1,2).

The central mission of the MGD is to support the translation of information from experimental mouse models to uncover the genetic basis of human diseases. As a highly curated and comprehensive model organism database, MGD provides web and programmatic access to a complete catalog of mouse genes and genome features including genomic sequence and variant information. MGD curates and maintains the comprehensive listing of functional annotations for mouse genes using Gene Ontology (GO) terms and contributes to the development of the GO content and structure (3). Finally, MGD curates and integrates comprehensive phenotype annotations wherein phenotypes are associated with genotypes using terms from the Mammalian Phenotype Ontology (4) and are represented with precise associations to relevant human diseases. These workflows enable detailed descriptions of the relevance and relationship of mouse models to human diseases (5).

MGD is a core component of an extensive set of genome informatics resources that collectively comprise the Mouse Genome Informatics (MGI) resource (http://www.informatics.jax.org). The MGI system includes the Gene Expression Database (6), the Mouse Tumor Biology Database (7) and the MouseCyc database of biochemical pathways (8), and provides the authoritative set of GO annotations for the laboratory mouse as a founding member of the GO Consortium (9). The MGI system overall provides an intensively integrated and accessible data resource representing the highest quality and most comprehensive consensus and experimental views of laboratory mouse as an experimental organism.

## IMPROVEMENTS

Recent improvements to MGD include integration of the latest mouse genome build (GRCm38), improved access to comparative and functional annotations for mouse genes and new uploads of phenotype data from the Sanger Institute Mouse Genetics Program (10) and the Europhenome (EuPh) Database (11). Major improvements have been made in access to strains and genes associated with Cre-recombinase constructs (used for cell- and tissue-specific expression) through new implementation of the CrePortal. A summary of the database content for MGD (September 2013) is given in Table 1.

**Table 1.** Summary of MGD content September 2013: stats from September 8 MGI public stats page

Current Stats	September 2013
Number of genes with protein sequence data	24 526
Number of mouse genes with human orthologs	17 092
Number of mouse genes with rat orthologs	17 811
Number of genes with GO annotations	25 495
Total number of GO annotations	257 164
Number of mutant alleles (cell lines only)	712 925
Targeted mutations	50 569
Number of mutant alleles in mice	34 538
Number of QTL	4714
Number of genotypes with phenotype annotation (MP)	48 862
Total number of MP annotations	254 327
Number of mouse models associated to human diseases	4130
Number of human diseases with one or more mouse models	1256
Number of references in the MGD bibliography	193 943

### Genome feature updates

MGD maintains a comprehensive catalog of genes, functional RNAs and other genome features as well as heritable phenotypes and quantitative trait loci (QTL). As described previously (12), the MGD genome feature catalog is generated by the integration of computational and manual genome annotations generated by NCBI, Ensembl and Vega/HAVANA. The genome coordinates for these features, and phenotypes were updated to the latest mouse genome assembly (GRCm38.p1). In addition to the standard web-based access to this catalog, the unified genome catalog is available via ftp in Generic Feature format (GFF) (ftp://ftp.informatics.jax.org/pub/mgigff/) to support the use of these data in bioinformatics applications.

Single nucleotide polymorphism (SNP) data in MGD were updated to NCBI dbSNP Build 137. With this update, all of the SNP and structural variation data from deep sequencing of 17 inbred strains of mice (13) are now integrated into MGD.

### New implementation and visualization of comparative genomic data

#### Incorporating external ortholog sets

**Figure 1.**
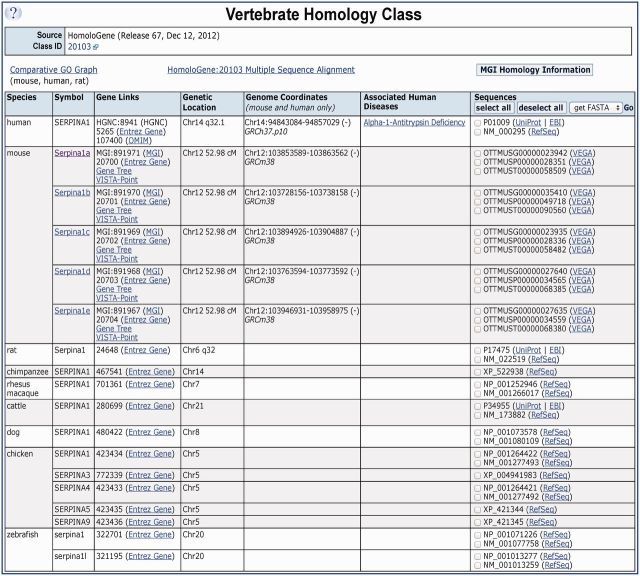
Homology Detail Page: Complete orthology set representation in MGD, derived from HomoloGene cluster 20103.

When the MGD was first created, it curated the mouse to human orthology set using sequence analysis tools and reporting sequence-based orthology as described in scientific publications. Orthology assertions form the basis for functional predications that exploit comparative relationships to infer function for mouse genes from experimentally determined knowledge in other organisms, particularly from experimental knowledge determined about human and rat genes. Although MGD has been incorporating orthology data from NCBI HomoloGene resource (14) for some years, these data were still represented within the context of MGD homology assertions and specifically restricted the relationships to a 1:1 assertion of orthology among mammals. In 2013, MGI implemented a many-to-many orthology paradigm to better reflect current understanding about the relationships between genes of these organisms. Although one-to-one orthology assertions between mouse/human/rat genes still hold for 98% of protein-coding genes, MGI can now more clearly represent cases such as *Serpina1a* (MGI:891971), where phylogenetic analysis shows five mouse genes and one human gene in the same orthology class (Figure 1). In addition, MGD is now importing orthology data from the HomoloGene, although revisions in the MGD schema support importation of orthology sets from any external resource.

#### Extending representation beyond mammals to include other vertebrate species

**Figure 2.**
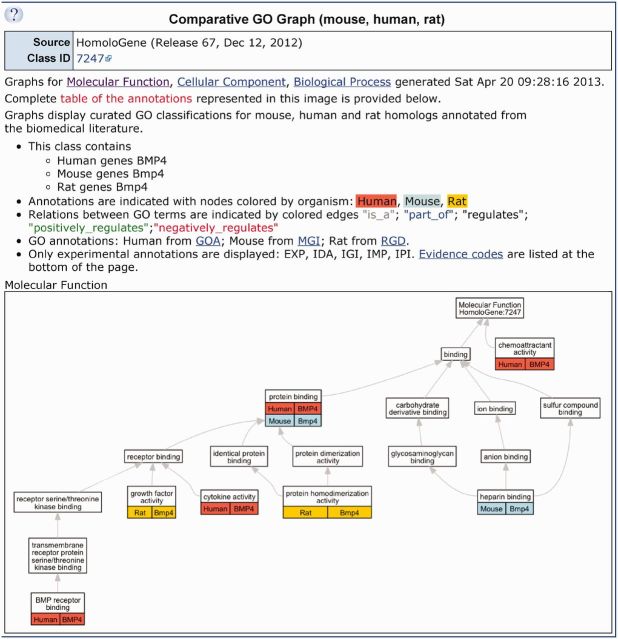
GO Comparative Graphs: Experimentally derived GO annotations available for human, rat and mouse for the Molecular Function domain. In the mouse annotation file, these human and rat annotations form the basis for Inferred from Sequence Orthology annotations for the mouse.

With the revision of the homology data, we extended the comparative data coverage from ‘mammalian’ to ‘vertebrate’ inclusion in MGD. The orthology data views, therefore, now include information from chicken and zebrafish genomes. As part of the updating process, we also now represent new graph views of comparative GO annotations for experimentally determined data from human, mouse and rat (Figure 2).

### Improvements in GO annotation completeness and visualization

MGD, a founding member of the GO Consortium, provides the comprehensive set of mouse functional annotations using the GO for mouse genes and gene products. MGD curators contribute to the development of the GO ontologies and participate in a variety of GO Consortium working groups including the PAINT phylogenetic analysis for functional annotation project (15). MGD expertise in curation of the biomedical literature provides the core experimental data used to infer function for orthologous genes in a broad comparative genomics context.

Following the revision of the MGD representation of vertebrate orthology, the GO team at MGD implemented new rules for loading of Inferred from Sequence Orthology annotations from other vertebrate species. These annotations are only generated when the contributing annotation is derived from experimental results in the specific organism [e.g. experimental data from human gene *BMP4* (UniProt record P12644) provides data through PMID 7811286 to assert through evidence code Inferred from Sequence Orthology that mouse *Bmp4* has inferred molecular function ‘BMP receptor binding’ (GO:0070700)].

### Major revisions in the CrePortal

Studies of cell-type and stage-specific gene regulation and function often use conditional mutagenesis, in which genes can be knocked out at specific sites in a spatial and temporal manner. To effectively use conditional mutagenesis, mice carrying an appropriate recombinase (e.g. Cre) construct are required for mating to mice bearing conditional-ready loxP-flanked genes.

The CrePortal (http://www.creportal.org) provides critical data about Cre constructs, including the driver, whether recombinase activity is inducible (and by what), strain availability through public repositories and publications describing conditional mutagenesis done using each Cre allele. Histological images, annotated with activity patterns, anatomical structures and ages, assayed defining Cre specificity can assist selection of optimal Cre-bearing strains for specific experiments. Whole slide viewing is available for some Cre lines, notably submitted by JAX Mice and from the Allen Institute of Brain Science. Access to Cre specificity data is critical in determining the best Cre-bearing strains for experiments, not only for knowledge about activity at the desired target (and its time/space distribution), but also for considering ‘off-target’ activity that may complicate interpretation of observed phenotypes. Through links to MGI (http://www.informatics.jax.org), phenotypic information for conditional genotypes that have been studied is also provided. As of September 2013, there were >2000 unique Cre alleles cataloged in the CrePortal.

**Figure 3.**
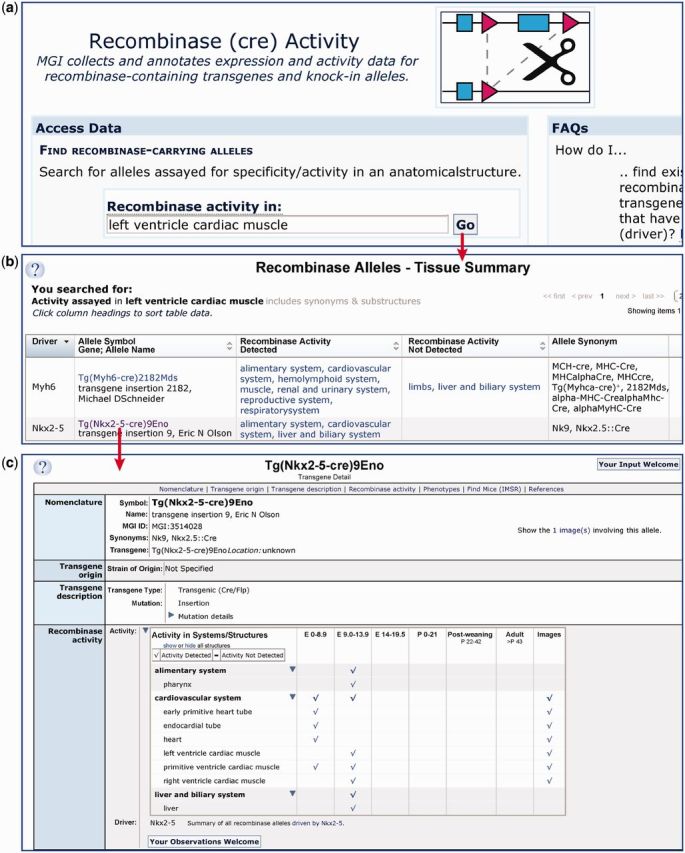
New features in the CrePortal: (**a**) Cre Search Form accessed at www.creportal.org or by choosing the Recombinase (Cre) icon box at www.informatics.jax.org. The term ‘left ventricle cardiac muscle’ has been entered in the search box. This field has an auto-complete feature that indicates both annotated terms (black type) and terms with no annotation (gray type). (**b**) Results from the above search for ‘left ventricle cardiac muscle’. Two Cre transgenes that show specific Cre activity in this tissue are returned. Formerly only systems searches were allowed; a search by ‘cardiovascular system’ returns 266 transgenes and knock-ins, most of which are not specific for left ventricle cardiac muscle (searches done 29 September 2013). (**c**) Overview of data for the Cre transgene, Tg(Nkx2-5-Cre)9Eno clicking through from the symbol on the Results page in (b). The recombinase activity matrix shows activity for this Cre transgene in structures/tissues assayed versus age. Note that anatomical systems have been toggled open to show specific tissues. For left ventricle cardiac muscle, one can visualize its activity by age distribution, and also note off-target embryonic expression of this Cre transgene in pharynx and liver.

Important new features have been implemented in the CrePortal in response to user comments (Figure 3). These include (i) the ability to search for Cre activity by specific tissue or structure such as ‘left ventricle cardiac muscle’ (formerly only searches by anatomical system were available, e.g. cardiovascular system); (ii) a summary matrix of Cre activity in structures/tissues assayed versus age, e.g. for left ventricle cardiac muscle, one can visualize its activity by age distribution; also note off-target embryonic expression in liver and pharynx; (iii) a new ‘Your Observations Welcome’ link for contributions of laboratory experience with particular Cre mouse strains, as many ‘facts’ about laboratory performance of Cre lines remain anecdotal; and (iv) a submission form for data and image files on new Cre strains, or additional data on existing strains.

### Integration of high-throughput phenotype data

MGI now includes high-throughput phenotyping data along with data submitted from laboratories and centers, and curated data from publications, providing comprehensive comparative phenotypes for mouse mutants. Current high-throughput data sets include those from the Wellcome Trust Sanger Institute (WTSI) (10) and the EuPh (11) database. This integration allows specific comparisons between different centers’ data interpretations and is a prelude to future MGI data integration from the International Mouse Phenotyping Consortium project sites and Data Coordination Center (16).

**Figure 4.**
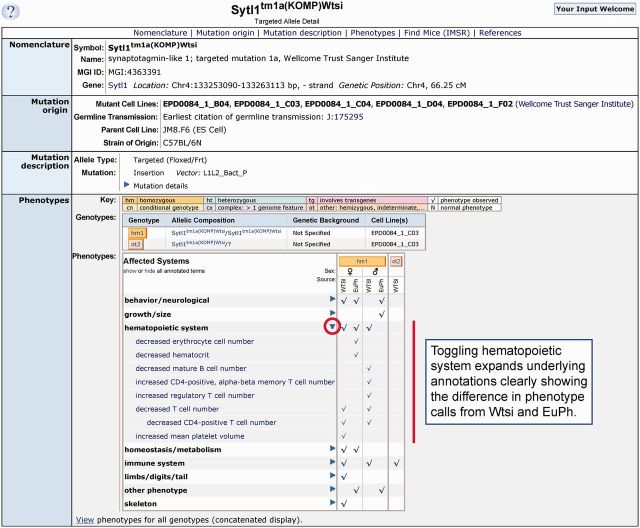
High-throughput Phenotype Data: This partial image of the phenotype detail page for the targeted allele Sytl1tm1a(KOMP)Wtsi illustrates the ability to compare high-throughput data results. In the ‘Phenotypes’ section, one is first presented with a colored key of the specific genotypes analyzed. This key corresponds to the columns of the phenotype table. In this example, the data for the ‘hm1’ homozygous females and males have been analyzed by both WTSI and EuPh databases. The hematopoietic system phenotype section has been expanded to show the clear differences in phenotype calls made on the same animal data sets by these two centers based on their differing analysis methods.

Importantly, this integration allows comparisons of knockout mouse phenotypes called from high-throughput phenotyping pipelines versus calls of the same mouse data by other analysis groups, or the same mouse knockouts phenotypically assessed by research groups specializing in particular phenotype assessments. In addition, MGI’s integrated phenotypic data allow comparison of these knockouts with other mutagenized alleles of a gene (e.g. ENU mutants, other genetically engineered constructs). Such comparison enables new hypotheses for gene function based on phenotype annotations for alleles of a gene and permits comparisons of phenotypic range when systematic phenotypic testing on defined genetic background is carried out. Figure 4 shows a portion of the MGD detail page for the knockout allele, *Sytl1^tm1a(KOMP)Wtsi^*. In the phenotypic table section, one can observe data from WTSI and the EuPh database. The specific annotations for the hematopoietic system are expanded to highlight the clear differences between phenotype calls made by the two groups using the same data. This difference in data interpretation is particularly relevant, as high-throughput data are generated, analyzed and integrated in different ways. MGD is committed to displaying these differences to inform researchers when seeking mice with particular characteristics, and as a differentiation between results from high-throughput pipelines using specific algorithms and statistical cut-offs in calling phenotypes versus traditional wet-bench or ‘low-throughput’ laboratories where more granular phenotypes and system studies produce focused sets of phenotypic analyses.

## COMMUNITY OUTREACH AND USER SUPPORT

MGD offers extensive resource support through online documentation, frequently asked questions, tutorials and Email and phone access to User Support staff.

User Support can be accessed by:

- World Wide Web: http://www.informatics.jax.org/mgihome/homepages/help.shtml
- Email access: mgi-help@jax.org
- Telephone access: +1 207 288 6445
- FAX access: +1 207 288 6132

The MGD User Support group also maintains and moderates two Email bulletin boards, MGI-LIST and MGI-TECHNICAL-LIST, for the mouse research community (http://www.informatics.jax.org/mgihome/lists/lists.shtml). Updates to the MGI resources are announced on the lists, and list subscribers can post questions for community discussion. MGI-LIST has > 2000 subscribers and an average of 60 posts/discussions per month. MGI-TECHNICAL-LIST is a smaller less active list that is geared toward computational access to MGI data.

## DATA SUBMISSION

Most of the data in MGD come from semi-automated curation of the peer-reviewed scientific literature and from collaborative/cooperative arrangements with large mouse-related data centers and repositories and other informatics resources. MGD also supports electronic data contributions directly from individual researchers. Any type of data that MGD maintains can be submitted as an electronic contribution. Other common types of submission include mutant and QTL mapping data. Each electronic submission receives a permanent database accession ID. All data sets are associated with their source, either a publication or an electronic submission reference. MGD reference pages provide links to associated data sets. Online information about data submission procedures is found at the following URL: http://www.informatics.jax.org/submit.shtml.

## SYSTEM OVERVIEW

The MGD database, software and hardware are organized into a front end, where the data are made available to the public, and a back end, where data are loaded and curated from various resources. In the past, the front end and back end shared a common database structure/schema, but in recent years, the two have been decoupled: the new front end is tuned for performance and web display, whereas the back end is designed to support data curation and integration. The front end database is highly denormalized and augmented by Solr/Lucene (http://lucene.apache.org/solr) indexes. During each weekly data release, data from the back end are migrated to the front end and Solr/Lucene indexes are populated. In addition to the significant performance improvements enjoyed by the user, the decoupling of the front and back ends also helps to limit and manage the ripple effects of changing either side.

### The front end–public data access

MGD provides free public access to its data in a number of ways; all are accessible from the main Web site: http://www.informatics.jax.org. The web interface, the software that provides the interactive searching and dynamic content on the web site, is the most commonly used access point. In addition to the simple keyword-based ‘Quick Search’ available on every web page, there are a variety of forms available for more involved queries including searches for Genes and Markers; Phenotypes, Alleles and Diseases; SNPs; and References. There are also vocabulary browsers for GO, Mammalian Phenotype Ontology (MP) and OMIM disease terms that support exploration of these vocabularies and access to all data in MGD annotated to each vocabulary term. Graphical views of the mouse genome and interactive genome browsing are supported by our Generic Genome Browser (GBrowse) instance, which was recently upgraded to GBrowse 2.X (http://gbrowse.informatics.jax.org/cgi-bin/gb2/gbrowse/mousebuild38/).

In addition to traditional query forms and data displays, MGD offers users several other ways to access data. The *Batch Query* tool (17) (http://www.informatics.jax.org/batch) supports bulk access to certain information about lists of genes. Users can upload identifiers from a wide variety of sources (MGI, Entrez, Ensembl, etc), have those IDs matched to genes in MGI and download specified information for those genes, e.g. genome coordinates and GO annotations. Results are available in HTML, tab delimited text or Excel format. Other parts of the web interface exploit this tool, allowing users to generate customized gene/feature summaries from query results.

Subsets of MGI data are also available through instances of BioMart and InterMine. These popular data warehousing systems offer interactive web interfaces as well as programmable APIs via Web Services. Our BioMart instance contains two data sets: mouse genes and genome features, and mouse developmental gene expression data. Our InterMine instance, called MouseMine (http://www.mousemine.org), contains the complete annotation ‘core’ of MGI, including the complete catalog of mouse genes, alleles and strains, plus annotations to the GO, Mammalian Phenotype Ontology and OMIM.

Finally, MGI offers access to a large set of regularly updated database reports via our FTP site (ftp://ftp.informatics.jax.org), and direct SQL access to a read-only copy of the database (contact MGI user support for an account). MGI User Support can also assist users in generating custom reports on request.

## CITING MGD

This article provides the general citation for use of the MGD resource. Please use the following format for citation when referencing data sets specific to the MGD component of the MGI resource: MGD, MGI, The Jackson Laboratory, Bar Harbor, Maine (URL: http://www.informatics.jax.org). [Type in date (month, year) when you retrieved the data cited.]
